# [2.2]Paracyclophane-Based Chiral Platforms for Circularly Polarized Luminescence Fluorophores and Their Chiroptical Properties: Past and Future

**DOI:** 10.3389/fchem.2020.00700

**Published:** 2020-10-29

**Authors:** Ken-ichi Sugiura

**Affiliations:** Department of Chemistry, Tokyo Metropolitan University, Hachioji, Japan

**Keywords:** carbazole, chirality, circularly polarized luminescence, cyclophane, multi-layer, pyrene

## Abstract

Quality of CPL fluorophore is defined by the vectors of electric dipole transition moment and imaginary magnetic dipole transition moment. The aim of this review is to introduce readers to a chiral moiety applicable to CPL studies focusing on chiral cyclophanes because the rigid cyclophanes are able to hold the vector directions of electric dipole transition moment and imaginary magnetic dipole transition moment.

## Introduction

Circularly polarized luminescence (CPL) is both an old and a new research field. As far as the author knows, the first CPL observation could be the spectrum for a chiral crystal of uranyl acetate (Na[UO_2_(CH_3_COO)_3_]) recorded at liq. He temperature in 1948 (Samoilov, 1948; Ferrari et al., 1959; Golovnya, 1963; Murata et al., 1979). Soon, researchers noticed the versatile applications of CPL and CPL science started attracting much attention. From the 1970's onwards, excellent reviews of the physics of CPL and the materials that show CPL have been published, reflecting the vast interest of scientists and engineers in CPL phenomena (Richardson and Riehl, 1977; Steinberg, 1978; Riehl and Richardson, 1986; Gussakovsky, 2008; Bradberry et al., 2014; Watanabe and Akagi, 2014; Sanchez-Carnerero et al., 2015; Zinna and Di Bari, 2015; Longhi et al., 2016; Han et al., 2018; Tanaka et al., 2018; Akagi, 2019). Basic theoretical background was already established in 1970' (Richardson and Riehl, 1977), however, the lack of a commercially available CPL apparatus hindered the progress of this research field and only a limited number of researchers who could make the apparatus were able to conduct research of CPL. The author likens CPL research in the 1980's to the *dark ages*.

In the 1980's, commercially available apparatus, i.e., *haute couture* apparatus, appeared in the market. Because of this, researchers, particularly synthetic chemists, gradually became accustomed to measuring CPL spectra. Today, many publications on CPL and related phenomena are available. CPL evolved into one of the fundamental physical properties to be reported when a researcher synthesizes a new chiral molecule, similarly to NMR, mass, IR, absorption, and conventional emission spectra.

One of the most important research topics in CPL science is how to improve the *g*-value of the fluorophore because the *g*-value is one of the most important indexes and/or the degree of CPL expressing the quality of a CPL fluorophore. Then, how does a researcher obtain high *g*-values? The *g*-value is defined by the ratio of the difference in intensity divided by the average total luminescence intensity, as follows:

$$\begin{array}{l} g=\;\frac{△l}{\frac{1}{2}l}=\;\frac{l_{L}-l_{R}}{\frac{1}{2}\left(l_{L}-l_{R}\right)}=4\frac{m}{\mu }\cos\theta_{\mu ,m} \end{array}$$

where μ and *m* are the vectors of electric dipole transition moment and imaginary magnetic dipole transition moment, respectively, and θ_μ, *m*_ is the angle between them (Zinna and Di Bari, 2015). The *g*-value is a function of μ and *m*. We can qualitatively estimate μ of the molecule; in other words, we can design a fluorophore having a large μ (Berova et al., 2007). However, the estimation of *m* is non-objective and it is too difficult to translate *m* into a molecular structure. Therefore, the development of good CPL fluorophores is literally a continuous process of *trial* and *error* and largely depends on a researcher's intuition.

The CPL fluorophores used in the early stage of this science were simple. For example, chiral ketone **1** is a milestone molecule studied in CPL science in the 1960's through the 1970's. Unfortunately, the low quantum yield of this chromophore prevented its application (Emeis and Oosterhoff, 1967). Along with the progress of asymmetric synthesis using chiral catalysts prepared from 1,1′-bi-2-naphthol (BINOL) **2** and related compounds, this molecule was also applied in CPL science. In contrast to the excellent results of asymmetric synthesis using **2**, the low quantum yield (Φ = 0.04) of **2** might have prevented the progress of CPL science (Hassan et al., 2015). In addition, the functionalization of **2** for CPL studies was usually carried out on the hydroxyl group(s) and the resultant ether linkage had a flexible conformation that led to ambiguous μ and *m* directions.

The aim of this review is to introduce readers to a chiral moiety applicable to CPL studies. Details of the physics of CPL and the proposed applications have been omitted because we already have many excellent reviews including those in this special issue. The author focuses on chiral cyclophanes for reasons discussed in the following section. Because the chemistry of chiral cyclophanes has a long and rich history, reports of these molecules are abundant (Hassan et al., 2020a). The author believes that a good CPL fluorophore having a high *g*-value could be obtained using the cyclophane skeleton. Actually, Tani and his coworkers reported one of the highest *g*-value for their carbazole-based cyclophanes, |1.3 × 10^−2^| (*vide infra*) (Tani et al., 2001, 2020). Furthermore, as most of the reported chiral cyclophanes lack chiroptical properties, the author also believes that a good CPL fluorophore already exists among them. In this review, the author also introduces reported chiral cyclophanes such as pyrenophanes, which show great potential in CPL science. This information is expected to benefit readers.

## Definition of Chiral Molecule, Classification of Chirality, and Cryptochirality IN CPL Science

A CPL fluorophore should be chiral. Because chirality is one of the most important concepts in chemistry, several definitions of a chiral molecule are known. The simplest definition is that a chiral molecule is a molecule having (a) carbon atom(s) to which four different groups are attached. For example, 5-ethyl-5-propyl-undecane **3**, in which one carbon has ethyl, propyl, butyl, and hexyl groups attached, is a chiral molecule (*vide infra*) (Rabjohn and Latina, 1954). The type of this chirality is called central chirality and the corresponding molecules belong to the *C*_1_ point group. In CPL science, molecules classified under this chirality type are few (*vide infra*).

**Figure 1:**
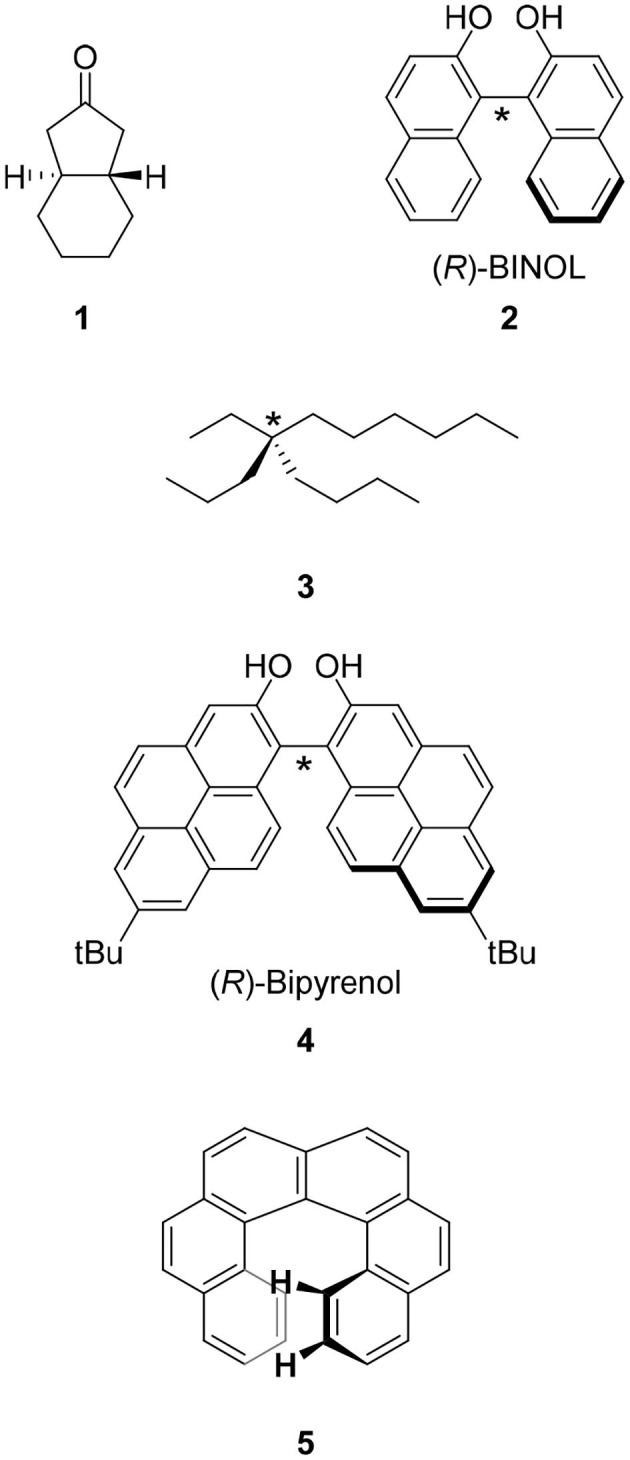
Molecular structures of CPL fluorophores studied in the early stage of this science. Molecular structures of one of the isomers of **1**–**5** are shown. The chiral center and chiral axes of 2-4 are indicated with asterisks.

Most of the chiral molecules studied in CPL science are not central chirality molecules. As described in the previous section, BINOL **2** is one of the most frequently used chiral groups in contemporary CPL science. This molecule has no carbon atom having four different groups. The chirality of BINOL is generated by the C-C bond between the two naphthalene moieties and this bond is called chiral axis (the chiral axis of **2** is denoted by “^*^” in Figure 1). This type of chirality is called axial chirality. In CPL science, a molecule must be able to emit light and therefore, arenes are the main category of molecules. The connection of two fluorescent arenes at the hindered position to restrict free rotation induces axial chirality. Based on this consideration, the author reported bipyrenol **4** having an improved quantum yield (Hassan et al., 2015).

Apart from central and axial chiralities is planar chirality. This type of chirality is found in chiral cyclophane, the main topic of this review. The detailed behavior of planar chirality is introduced in the following section using cyclophanes as example. We also have helical chirality represented by helicene, i.e., π-expanded arenes. However, such helicenes as [6]helicene **5** show extremely low quantum yields (Birks et al., 1976). The CPL science based on helical chirality and helicenes is an issue for the future.

Next, the author would like to refresh readers' minds regarding *cryptochirality*. An important example is the optical rotation of chiral molecule **3**. Optical rotation measurement was carried out for optically pure 3 but the spectra were silent (Wynberg et al., 1965; Wynberg and Hulshof, 1974) because the electrons of the four structurally similar substituents induced negligible perturbation of the electric fields. Later, the term *cryptochirality* was coined to describe this phenomenon. This phenomenon is applicable to CPL science, namely, even if we design a chiral fluorophore, the molecule would not always emit CPL. Revealing the reason would be tantamount to revealing the molecular design of a CPL fluorophore. Therefore, the author sincerely hopes that researchers report negligible CPL behaviors of chiral fluorophores. These “undesirable” information could open doors to the synthetic strategy of a CPL fluorophore.

## Chiralities of Substituted [2.2]Paracyclophanes

**Figure 2:**
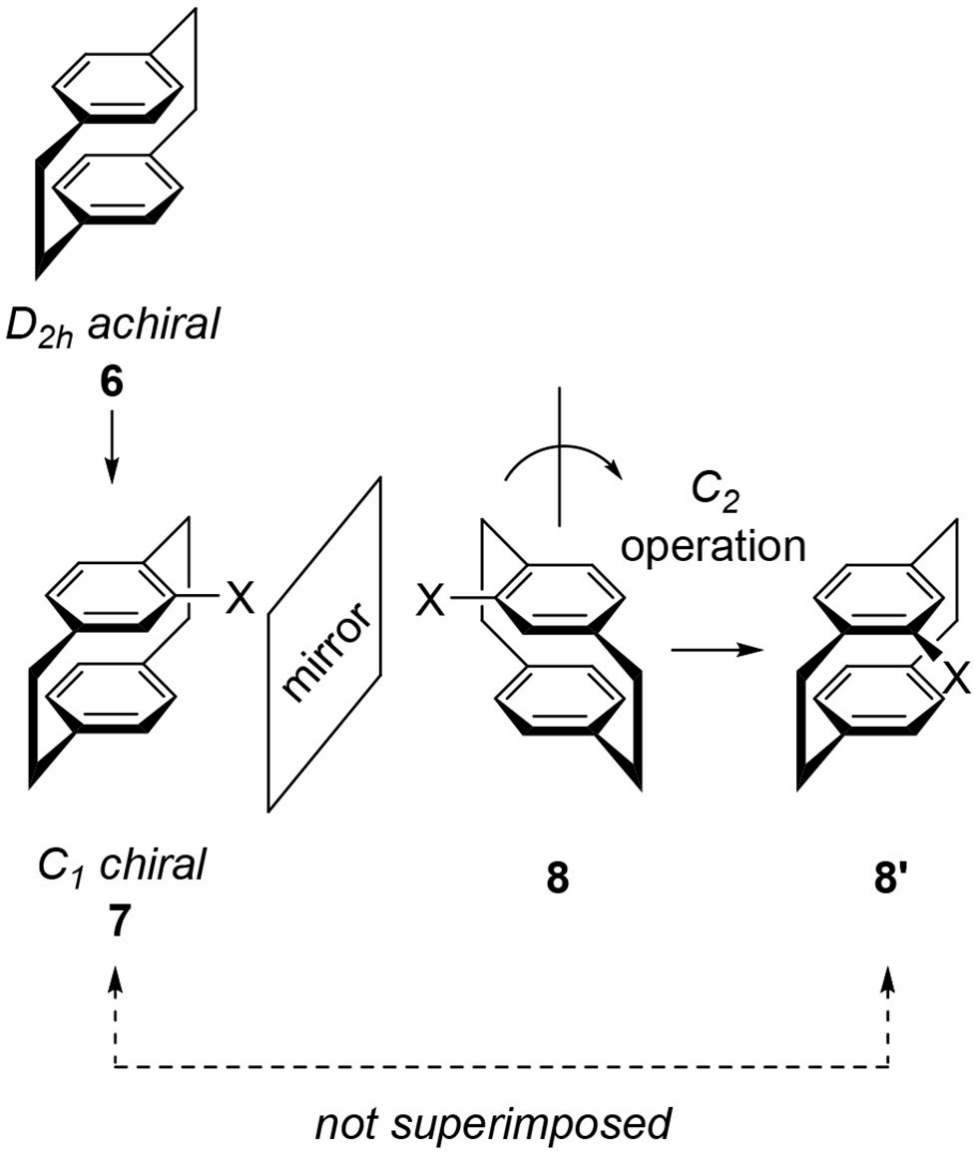
Molecular structures of [2.2]paracyclophane **6** and its monosubstituted derivative **7**. **8** or **8′** are the mirror image of **7** and **8**/**8′** is different from **7**. Therefore, **7** and **8** are enantiomers.

“Cyclophane” is the general term for arenes having cyclic moieties and various types of molecules are known (Vögtle, 1993; Gleiter and Hopf, 2004). In this review, the author only deals with stacked arene dimers connected by ethylene bridges and their related compounds. A representative example is achiral [2.2]paracyclophane **6**, a symmetrical molecule classified into the *D*_2_*h* point group (Figure 2). The introduction of substituent *X* onto the benzene ring of **6** produces monosubstituted cyclophane **7**, a chiral molecule. Here we examine this chirality by reflecting **7** on a mirror to give image **8**. To compare the generated molecular shape of **8** with the molecular shape of original **7**, a rotation operation is carried out to produce another image **8′**. Obtained **8′** is different from **7** and therefore, **7** and **8** are enantiomers. Monosubstituted **7** is chiral and its point group is *C*_1_.

**Figure 3:**
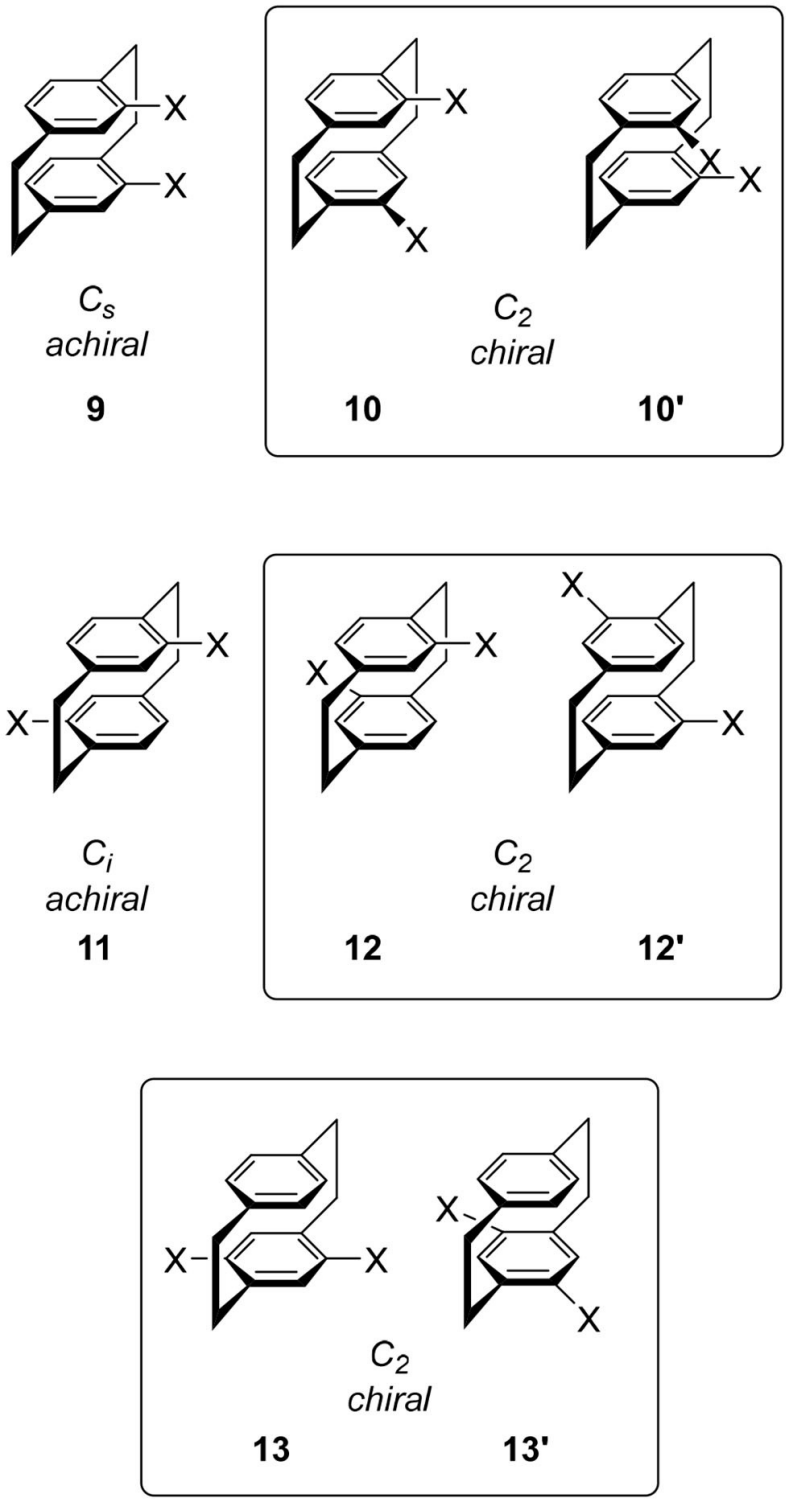
Molecular structures of disubstituted [2.2]paracyclophane derivatives **9**–**13**.

The disubstitution of **6** also induces chirality. Recently, an excellent review of the substitution manner of [2.2]paracyclophane was published and a systematic consideration of the chirality of the disubstituted molecule was introduced (Hassan et al., 2020b). To avoid duplication, important derivatives in CPL science are introduced here. When we introduce one substituent each onto the two benzenes, we obtain four types of isomers **9**, **10**, **11**, and **12**. The point groups of **9** and **11** are *C*_*s*_ and *C*_*i*_, respectively, and these molecules are not chiral. In contrast, the point groups of **10** and **12** are *C*_2_, i.e., these molecules are chiral. The structures of the corresponding enantiomers **10′** and **12′** are shown in Figure 3. The introduction of two substituents on one benzene ring also forms a chiral cyclophane, e.g., **13**, which belongs to the *C*_2_ point group generating enantiomer **13′**. This substitution manner is the key to multilayered chiral cyclophanes (*vide infra*).

## [2.2]Paracyclophane-Based CPL Fluorophore

As discussed above, monosubstituted **7** and disubstituted **10**, **12**, and **13** are chiral [2.2]paracyclophanes. When we introduce fluorescent group *X* onto these cyclophanes using, e.g., Pd-catalyzed coupling reactions, candidates for CPL are generated. In this review, three important examples are introduced, as follows.

**Figure 4:**
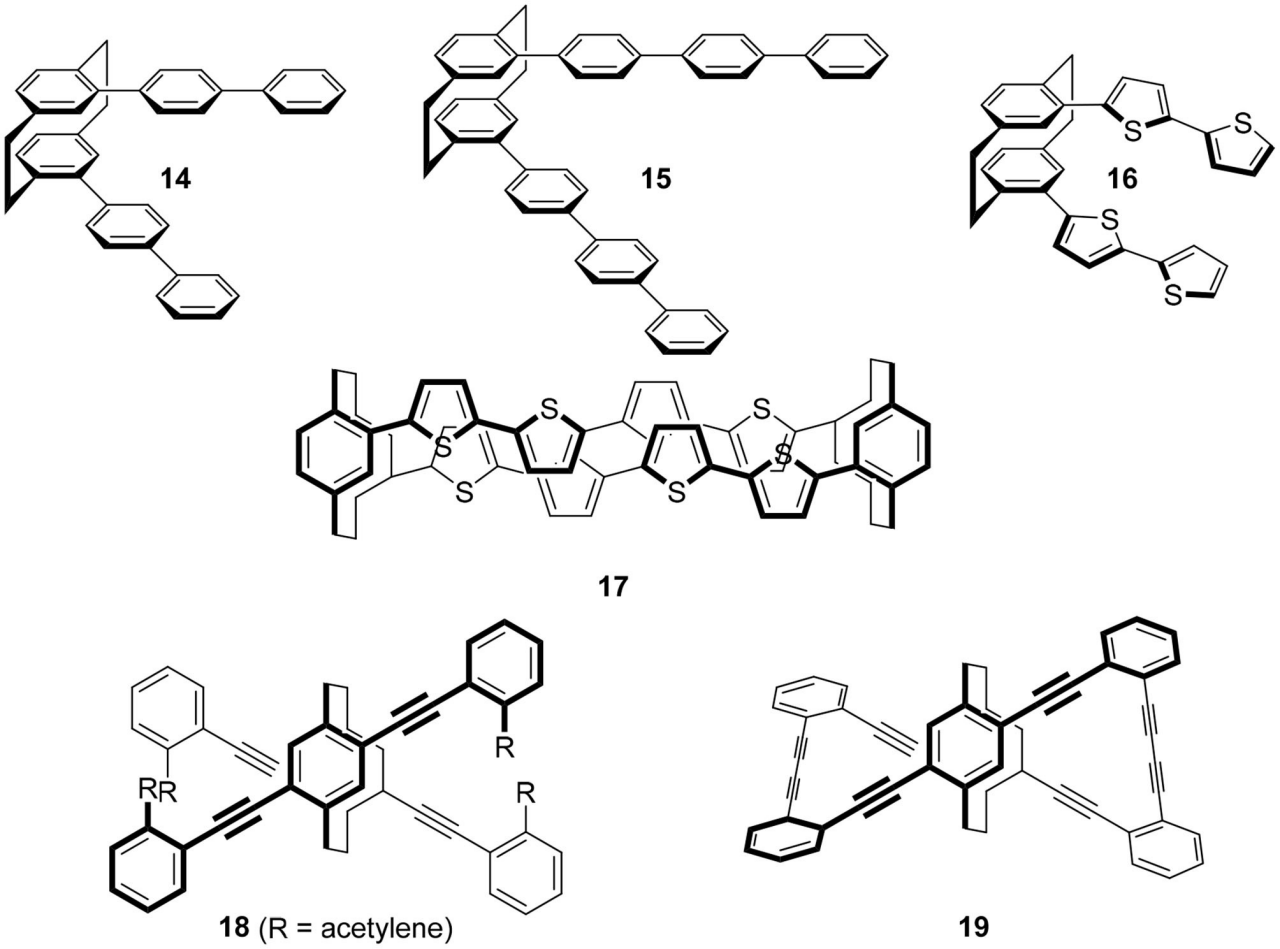
Molecular structures of CPL fluorophores **14–19** based on [2.2]paracyclophane skeleton. The substituents were omitted for clarity.

The molecule to be introduced first is the simplest yet most informative example (Figure 4). Hasegawa and coworkers focused on *para*-phenylene fluorophores and introduced these fluorophores onto chiral **10** (*X* = Br) by the Suzuki coupling reaction to produce **14** and **15**, respectively (Ishioka et al., 2019). The two terphenyls in **14** and the two quaterphenyls in **15** are arranged in a chiral manner, and the termini of these fluorophores interact strongly through the [2.2]paracyclophane skeletons. Their quantum yields are dependent on the introduced para-phenylenes, i.e., Φ = 0.20 and 0.64 for **14** and **15**, respectively. Reflecting these chiral alignments, the expected CPL spectra were observed and *g* = |4.2 × 10^−3^| (at 381 nm) and *g* = |1.5 × 10^−3^| (at 385 nm) for **14** and **15**, respectively.

Although the publication years of the papers are back and forth, this strategy has produced various CPL fluorophores by replacing the *X* of **10**. For example, the replacement of *para*-phenylenes of **14** and **15** with oligothiophenes (OTs) gave **16** because OTs are one of the most important molecules in photochemistry. Then, the terminus of this molecule was capped with the [2.2]paracyclophane derivative to afford **17** (Hasegawa et al., 2017). Although they did not measure CPL of these molecules, the clear CD spectra of these molecules have enhanced expectations of their potential applications.

Other than thiophene, various π-systems were introduced as *X* of **10** and characteristic chiral molecules were synthesized. Morisaki's group introduced acetylenes using the Sonogashira coupling, **18** (Morisaki et al., 2014). Acetylenes offer the advantage of further functionalization under mild reaction conditions. Subsequently, they carried out the Glaser-type diacetylene formation reaction, which produced *Escher's trompe l'œil* style cyclic molecule **19**. Although the quantum yield was moderate, Φ = 0.45, its *g*-value was extremely high as a small organic molecule, |1.1 × 10^−2^|. Presumably, the rigidity of cyclophane and the strong interaction of the chromophore via the cyclophane unit would contribute to these chiroptical properties.

## Multilayered Cyclophane

In the following two sections, the author introduces old molecules synthesized in the 1970's through the 1980's. Of course, no synthetic chemists measured the CPL spectra of those molecules at that time. However, the author believes that the molecule(s) shown here could become leading compound(s) in future CPL science, inspiring breakthroughs.

**Figure 5:**
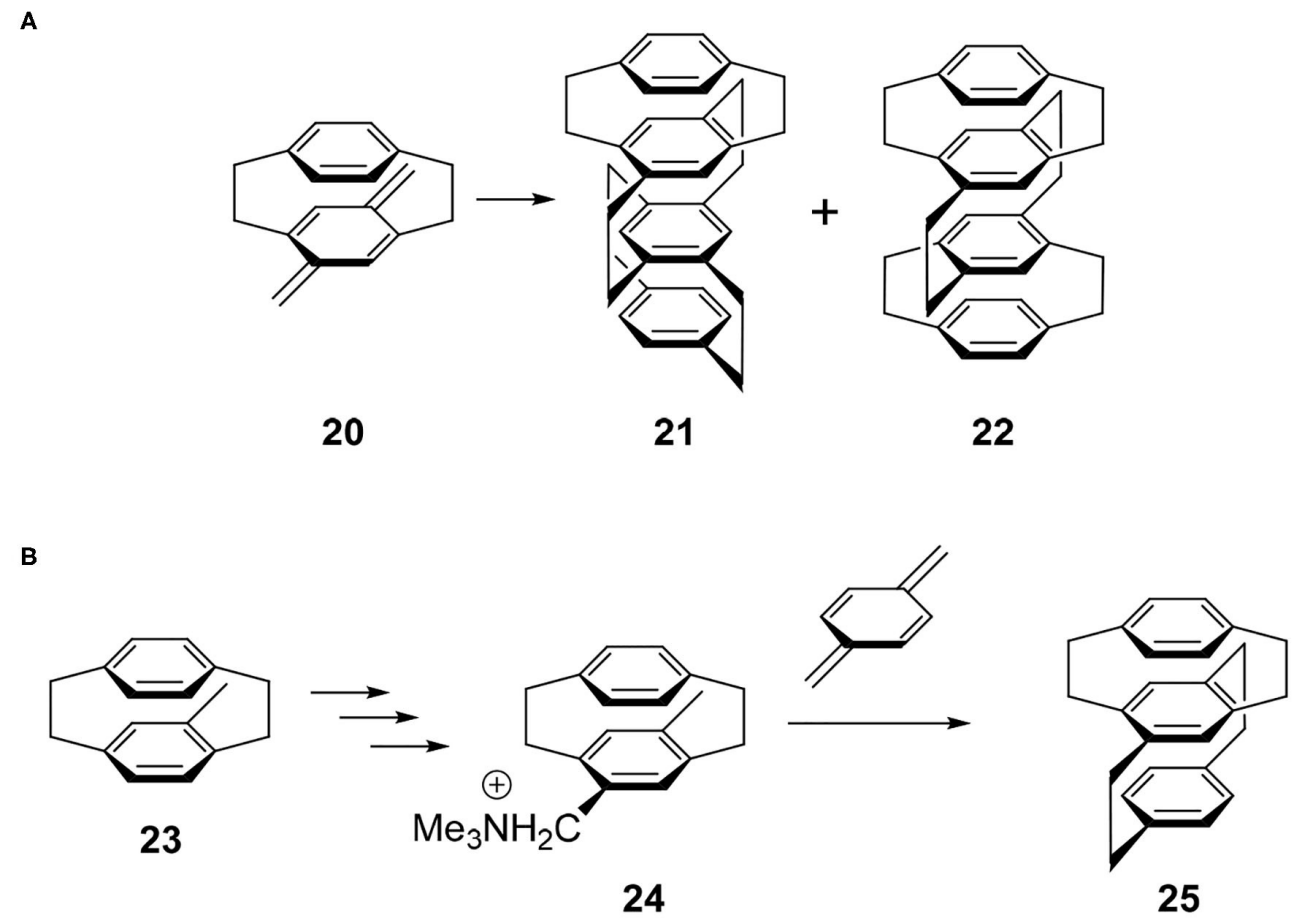
**(A)** Synthesis of multilayered [2.2]paracyclophane derivatives **21** and **22**. **(B)** Stereoselective synthesis of four-layered cyclophane.

The synthetic study of multilayered cyclophanes is one of the most exciting research topics in the history of cyclophanes (Misumi, 1983). The motivation of this chemistry was to reveal the through-space π-π interaction and ring current effects between the arenes. As far as the author knows, the first multilayered [2.2]cyclophane was a four-layered cyclophane (Longone and Chow, 1964, 1970). This molecule was prepared by the dimerization reaction of thermally generated *para*-xylylene intermediate **20** and this reaction afforded a mixture of **21** and **22** (Figure 5A). Later, Otsubo and Misumi performed fractional crystallization to give readily and sparingly soluble fractions. Comparing the spectra with those of authentic samples synthesized from structurally well-confirmed precursors, the readily soluble solid was determined to be **21** belonging to the *D*_2_ point group, a chiral molecule focused in this review. Product **21** should be a thermodynamically controlled product to avoid steric repulsion of the bridges, whereas **22** could be a kinetically controlled product.

Because above-mentioned chiral molecule **21** was racemic, Yamamoto and Nakazaki achieved a stereoselective synthesis using a known optically pure cyclophane precursor (Nakazaki et al., 1977). For example, optically pure **23** was converted into **24** and the subsequent coupling reaction with *para*-xylylene afforded optically pure three-layered compound **25** (Figure 5B). Along with this compound, they reported the CD spectra of a series of optically pure cyclophanes ranging from two- to six-layered compounds.

**Figure 6:**
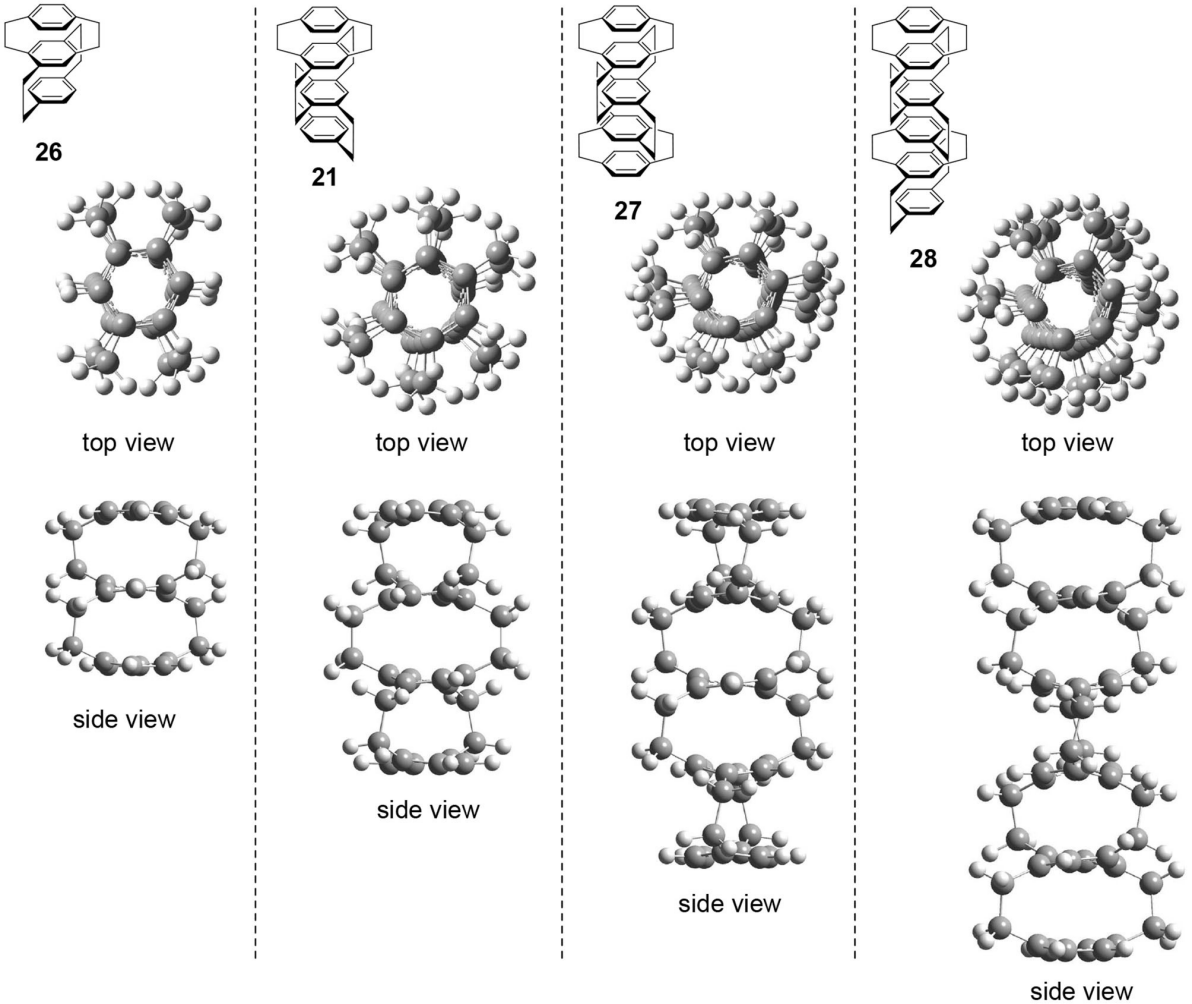
Top and side views of theoretically optimized structures of multilayered [2.2]paracyclophane derivatives **26**, **21**, **27**, and **28** [B3LYP/6-31G(d)].

The author performed preliminary theoretical studies of these multilayered chiral cyclophanes, i.e., three-layered **26**, four-layered **21**, five-layered **27**, and six-layered **28**. The optimized structures are shown in Figure 6. First of all, the author points out that all of these molecules belong to the *D*_2_ point group regardless of the odd or even number of benzenes. The obtained structures reproduce the experimental result, i.e., the twisted boat conformation of the interior benzenes. The top views of the molecules suggest that the stacked benzenes take a screw alignment similar to the helical assembly of a chiral discotic liquid crystal that shows CPL (Wu et al., 2017). Therefore, CPL studies of multilayered cyclophanes could be important to reveal the molecular design strategy of the chromophore.

## Cyclophanes Using Arenes Other Than Benzene: Carbazolophane and Pyrenophane

**Figure 7:**
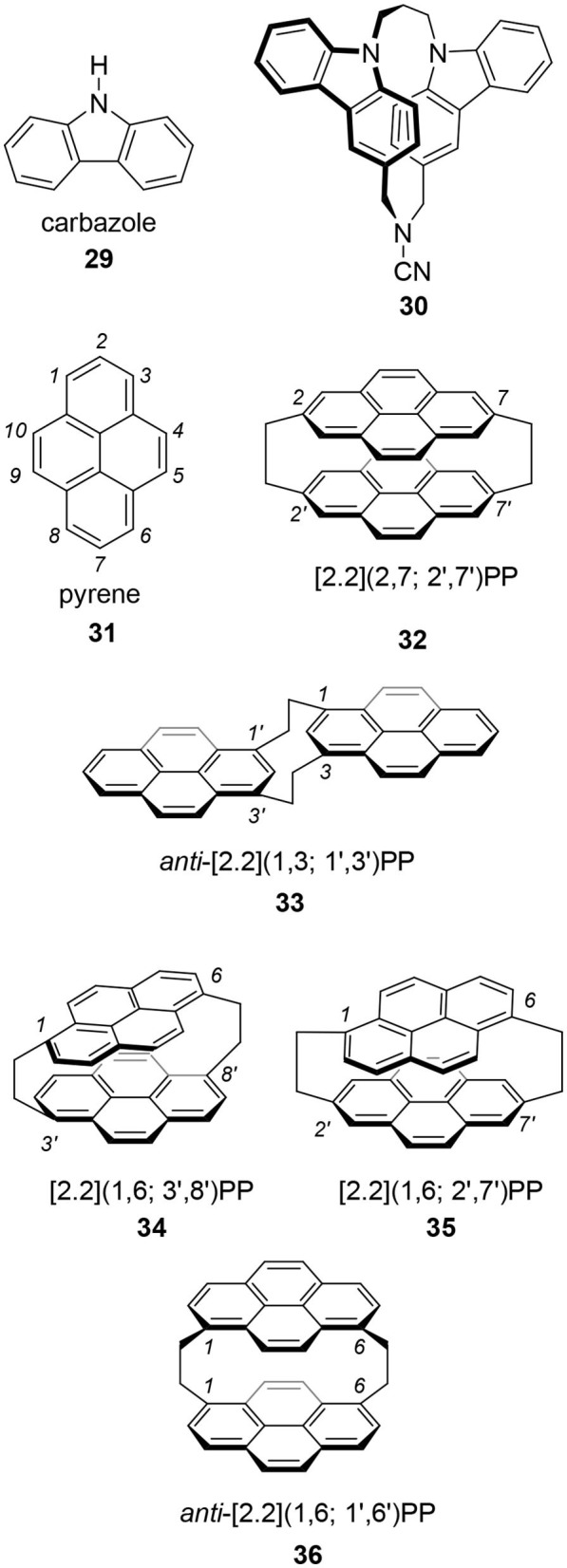
Cyclophane derivatives using carbazole **29** and pyrene **31**.

The chiral cyclophanes introduced in the previous sections show moderate *g*-values. However, their quantum yields are not so high for practical applications. If we replace the benzene moieties of **6** with fluorescent arenes, what will happen? As described, the author replaced the naphthalene of **2** with pyrene, a standard molecule in photochemistry, and good CPL fluorophore **4** was obtained (Hassan et al., 2015). A similar strategy could be applicable to other molecular systems (Figure 7).

Tani's group focused on carbazole **29** as the best candidate because carbazole is one of the widely applied molecules in photoelectronic technologies (Tani et al., 2001, 2020). They prepared chiral carbazolophane **30** and its enantiomer. Although its quantum yield was low, Φ = 0.047, its *g*-value was extremely high as a small organic molecule, |1.3 × 10^−2^|. That they used propylene ([3.3]) as the linkage suggests that a shorter [2.2] linkage is not always needed for a good CPL fluorophore showing a high *g*-value.

Pyrene **31** is one of the most popular fluorophores in chemistry (Winnik, 1993; Casas-Solvas et al., 2014). Therefore, CPL research of a pyrene-based cyclophane, pyrenophane (PP), should be interesting. Using two ethylene bridges, i.e., [2.2] linkage, various [2.2](n,m; n′,m′)pyrenophanes are possible, where n, m, n′, and m′ are the position numbers of each pyrene. In the 1970's, several isomers were reported. At that time, the motivation for PP research was the model of a pyrene excimer. In 1975, Misumi reported the first PPs, i.e., [2.2](2,7; 2′,7′) **32** (Umemoto et al., 1975b; Staab and Kirrstetter, 1979) and *anti*-[2.2](1,3; 1′,3′) 33 (Umemoto et al., 1975a). Later, Misumi synthesized other isomers: [2.2](1,6; 3′,8′) 34, [2.2](1,6; 2′,7′) **35**, and [2.2](1,3; 1′,3′) **36** (Kawashima et al., 1978). Among these five isomers, **34** and **35** are chiral and their point groups are *D*_2_ and *C*_2_, respectively. It is unfortunate that few photophysical properties of these molecules are known because the synthesis of these molecules is time-consuming and difficult. However, the author hopes a revised synthetic scheme would be developed using contemporary reactions to provide **34** and **35** for chiroptical studies in the future.

## Conclusion

In this short review, the author introduced cyclophane-based CPL fluorophores along with chiral cyclophanes having potential as a CPL fluorophore. Contemporary research of CPL has just started. Along with the basic photophysical chemistry achievements introduced in the literatures shown in introduction section, fundamental research topics, e.g., the relationship between the chirality and light (Ayuso et al., 2019), are also ongoing.

Experiments for known chiral fluorophores is just as important as the synthesis of a new fluorophore for CPL study. Cyclophane derivatives are favorable for CPL science because these rigid molecules are able to hold the vector directions of electric dipole transition moment μ and imaginary magnetic dipole transition moment *m*. Using known synthetic methods of cyclophanes, researchers could arrange the orientations of fluorophores as desired. Currently, the relationship between bridge length and CPL and/or chiroptical properties is unknown. However, it should be an important parameter for molecular design. Past synthetic methods of cyclophanes could also solve these problems.

## Author Contributions

The author confirms being the sole contributor of this work and has approved it for publication.

## Conflict of Interest

The author declares that the research was conducted in the absence of any commercial or financial relationships that could be construed as a potential conflict of interest.
